# Juruin: an antifungal peptide from the venom of the Amazonian Pink Toe spider, *Avicularia juruensis*, which contains the inhibitory cystine knot motif

**DOI:** 10.3389/fmicb.2012.00324

**Published:** 2012-09-10

**Authors:** Gabriela Ayroza, Ivan L. C. Ferreira, Raphael S. R. Sayegh, Alexandre K. Tashima, Pedro I. da Silva

**Affiliations:** ^1^Laboratório Especial de Toxinologia Aplicada, Instituto ButantanSão Paulo, Brazil; ^2^Coordenadoria de Controle de DoençasSão Paulo, Brazil; ^3^Departamento de Ciências Exatas e da Terra, Universidade Federal de São PauloDiadema, Brazil

**Keywords:** Juruin, juruentoxins, *Avicularia juruensis*, inhibitory cystine knot motif, Theraphosidae venom, antimicrobial peptides

## Abstract

The aim of this study was to screen the venom of the theraposid spider *Avicularia juruensis* for the identification of antimicrobial peptides (AMPs) which could be further used as prototypes for drug development. Eleven AMPs, named juruentoxins, with molecular weight ranging from 3.5 to 4.5 kDa, were identified by mass spectrometry after the soluble venom was separated by high performance liquid chromatography. Juruentoxins have a putative inhibitory cystine knot (ICK) motif, generally found in neurotoxins, which are also resistant to proteolysis. One juruentoxin that has 38 amino acid residues and three disulfide bonds were characterized, to which we proposed the name Juruin. Based on liquid growth inhibition assays, it has potent antifungal activity in the micromolar range. Importantly, Juruin lacks haemolytic activity on human erythrocytes at the antimicrobial concentrations. Based on the amino acid sequence, it is highly identical to the insecticidal peptides from the theraposid spiders *Selenocosmia huwena, Chilobrachys jingzhao*, and *Haplopelma schmidti* from China, indicating they belong to a group of conserved toxins which are likely to inhibit voltage-gated ion channels. Juruin is a cationic AMP, and Lys22 and Lys23 show maximum positive charge localization that might be important for receptor recognition. Although it shows marked sequence similarity to neurotoxic peptides, Juruin is a novel exciting molecule with potent antifungal activity, which could be used as a novel template for development of drugs against clinical resistant fungi strains.

## Introduction

While combinatorial libraries have been widely used for generating diverse synthetic chemical compounds (Martin et al., 1995; Kirkpatrick et al., 1999), spider venom is a library with naturally selected, biologically active peptides with high target specificity. Therefore, spider toxins have been increasingly used as pharmacological tools and prototypes for drug development. From an evolutionary perspective, spiders belong to a very ancient and diversified group of arthropod with more than 40,700 described species, distributed in approximately 109 families, which makes them the most abundant terrestrial predators (Escoubas and Rash, 2004; Herzig et al., 2011).

The molecular diversity of spider venom is estimated in over 12 million biological active peptides. These toxins show different biologically activities, some of which have evolved into highly selective inhibitors of cell receptors (e.g., voltage-gated K+ ion channels) (Escoubas and Rash, 2004). The mix of several types of cell proteins and toxin peptides may act synergistically against their target, causing the venom noxious effects on its prey (Herzig et al., 2011). Moreover, the structure, function, and pharmacology of specific ion channels have been revealed by the mechanism of action of several spider toxins. Additionally, toxins binding selectivity and neuromodulatory effects could be used in the treatment of neurodegenerative disorders, such as epilepsies, Alzheimer and Parkinson's disease (Estrada et al., 2007; Saez et al., 2010). Advances over the past decades in mass spectrometry and molecular biology methods have allowed the characterization of genes related to such peptide toxins, which shed new light on the molecular diversity and evolution of these living combinatorial libraries (Corzo and Escoubas, 2003; Escoubas and King, 2009).

Spider toxins diversity is mainly based on small sized disulphide-rich peptides, which are suggested to fall into a limited number of structural patterns. Toxic peptides mainly conform to the Inhibitory Cystine Knot (ICK) motif, with a disulfide bond pairing of CI–CIV, CII–CV, CIII–CVI (Escoubas and Rash, 2004). It has been proposed that ICK toxins from spider venom have evolved from β-defensin gene duplications, diversification and further neofunctionalization (Fry et al., 2009). Defensins are among the most widely distributed innate immunity–related antimicrobial peptides (AMPs). In fact, cysteine-knotted (ICK-related) structural dissection revealed a minimal structure with potent antimicrobial activity (Vila-Perelló et al., 2005), and even highly specific arachnid neurotoxins, which bind to insect voltage-gated ion channels, have demonstrated antimicrobial activity (Redaelli et al., 2010). Convergently, Drosomycin, a β-defensin that acts in the immune response of *Drosophila melanogaster*, inhibits Na^2+^ channels in a manner similar to those of scorpion neurotoxins (Cohen et al., 2009). Therefore, the study of spider venom, a rich source of toxic peptides which exhibit the ICK motif might reveal novel exciting prototypes for antimicrobials design (Escoubas and Rash, 2004).

Accordingly to the ArachnoServer 2.0 <http://www.arachnoserver.org/>, a database of toxic proteins from spiders venom, 916 different peptides from 85 species have been described so far (Herzig et al., 2011). Possible new peptides and toxins have been revealed by the combination of mass spectrometry and transcriptomic analysis, as well as screening the venom for desired properties. However, knowledge about the composition of the venom of many spider species remains very poor (Diego-García et al., 2010).

**Figure 1:**
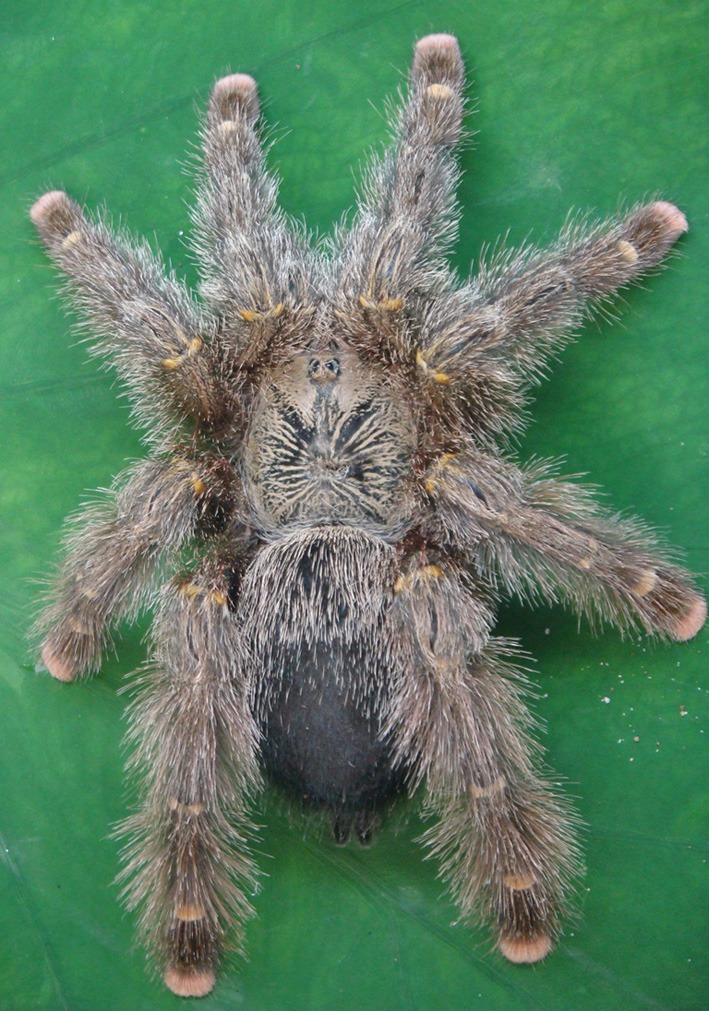
**Adult female *Avicularia juruensis* (Theraphosidae, Mygalomorphae).** Photo: Ayroza, G.

Here, we started to investigate the venom of the arboreal species *Avicularia juruensis* (Figure 1). The *Avicularia* genus comprises 13 species, endemic from regions in Central and South America, with at least three species threatened by habitat loss and illegal trafficking (Bertani and Fukushima, 2009). The Amazonian Pink Toe spider, *Avicularia juruensis* (Mello-Leitão, 1923), is a tarantula considered as an extremely docile species and not toxic to human being. Together with its stunning color and size, tarantulas from the *Avicularia* genus are one of the animals that are most often chosen as exotic pets. Even though the Amazonian Pink Toe spider is widely known, there are no studies available about its venom composition. Hence, the aim of this work was to explore the venom composition from *A. juruensis*, particularly to search novel antimicrobial compounds. This study is to the best of our knowledge the first venom analysis from the Brazilian spider *A. juruensis* (Amazonian Pink Toe), which resulted in the characterization of novel ICK toxins named juruentoxins.

## Materials and methods

### Bacterial strains

Fungal and bacterial strains were obtained from various sources. *Escherichia coli* SBS363 and *Micrococcus luteus* A270 were from the Pasteur Institut, Paris; *Candida albicans* (MDM8) was from the Department of Microbiology from the University of São Paulo, Brazil; *E. coli* ATCC 25922, *Pseudomonas aeruginosa* ATCC 27853, *Staphylococcus aureus* ATCC 29213, and *S. epidermidis* ATCC 12228 were from the American Type Culture Collection (ATCC). The following human clinical yeast isolates, which can be agents of candidiasis disease, obtained from the Oswaldo Cruz Institute, Brazil, were also used: *Candida krusei* IOC 4559, *C. glabrata* IOC 4565, *C. albicans* IOC 4558, *C. parapsilosis* IOC 4564, *C. tropicalis* IOC 4560, and *C. guilliermondii* IOC 4557. The filamentous fungi *Aspergilus niger* and the entomopathogenic fungus *Beauveria bassiana* were isolated from a mummified spider.

### Animals

The spiders (*Avicularia juruensis*, a tarantula of the Theraphosidae family) were kept alive in the biotherium of the Center for Applied Toxinology, of the Butantan Institute (São Paulo, Brazil) (Figure 1). These animals were collected under license Permanent Zoological Material no.11024-3-IBAMA and Special Authorization for Access to Genetic Patrimony no.001/2008.

### Venom fractionation and juruin isolation

In short, adult spiders were electrically stimulated for venom. *A. juruensis* crude venom was resuspended in 0.1% aqueous trifluoroacetic acid containing 10% acetonitrile (CH_3_CN), and the insoluble material was removed by centrifugation at 14,000×*g* for 5 min. The supernatant was used directly for HPLC separation. The diluted venom was fractionated using a reverse-phase semipreparative C18 column (Jupiter, 10 × 250 mm) equilibrated in 0.05% trifluoroacetic acid and eluted with a linear gradient from solution A [0.05% (v/v) trifluoroacetic acid in water] to 80% solution B [0.10% (v/v) trifluoroacetic acid in acetonitrile] run for 60 min at a flow rate of 1.5 ml/min. Effluent absorbance was monitored at 225 nm. Fraction with antimicrobial activity (Juruin) was further purified using a distinct gradient from 30 to 40% solution B run for 60 min in the same system. The purity of the peptide was ascertained by a symmetrical peak on the HPLC system, amino acid sequencing, and mass spectrometry analysis.

### Reduction and alkylation

Freeze-dried purified protein was dissolved (1 mg/ml) in denaturant buffer [6 M GdmCl (guanidinium chloride), 0.25 M Tris/HCl and 1 mM EDTA, pH 8.5]. To the mixture, 20 μl of 2- mercaptoethanol (Sigma) was added, followed by vortex-mixing and incubating at 37°C for 2 h. After incubation, 100 μl of 4-vinylpyridine was added to the solution, followed by incubation at room temperature (26°C) for 2 h. It was then subjected to RP-HPLC and the protein was eluted. The reduction and alkylation of the protein were confirmed by checking the mass using MALDI-TOF-MS. The reduced and alkylated protein was fragmented by enzymatic cleavage with trypsin (Boehringer Mannhein). Tryptic peptides were sequenced using tandem mass spectrometry (MS/MS) in a Q-TOF Ultima API (Micromass) spectrometer operating in positive ion mode. The sequence was deposited in UniProt (http://www.uniprot.org/) under accession number B3EWQ0.

### Mass spectrometric analysis

The samples containing the peptide fragments (0.5 μ l) were spotted onto the sample slide and dried on the bench and crystallized with 0.5 μ l of matrix solution [5 mg/ml (w/v) CHCA (α-cyano-4-hydroxycinnamic acid), in 50% acetonitrile and 0.1% TFA] (Sigma). The samples were analyzed on an Ettan MALDI-ToF/Pro spectrometer (Amershan Biosciences) operating in reflectron mode. To determine the amino acid sequence of peptides, the doubly charged ions were subjected to “*de novo*” sequencing in a Q-TOF Ultima API (Micromass) spectrometer operating in positive ion mode. The spectrum was analyzed, and the “y” and “b” fragments were used to elucidate the primary structure of the molecule.

### Antimicrobial assays

During the purification procedure, the antimicrobial activities of the samples were monitored by liquid growth inhibition assays using the Gram-negative bacteria *Escherichia coli* SBS363 and Gram-positive bacteria *Micrococcus luteus* A270 that were cultured in poor broth nutrient medium (PB: 1.0 g peptone in 100 mL of water containing 86 mM NaCl at pH 7.4; 217 mOsM), whereas yeast strain *Candida albicans* MDM8 was cultured in poor dextrose broth (1/2 PDB: 1.2 g potato dextrose in 100 mL of H_2_O at pH 5.0; 79 mOsM) used at half-strength as previsouly described (Hetru and Bulet, 1997; Bulet, 2008). Determination of antimicrobial activity was performed using 5-fold micro titer broth dilution assay in 96-well sterile plates at a final volume of 100 mL. Mid-log phase culture was diluted to a final concentration of 1 × 10^5^ colony forming units/mL. Dried fractions were dissolved in 200 μL of ultrapure water and 20 μL applied in to each well and added to 80 μL of the bacterium/yeast dilution. The fractions were tested in duplicate. 100 μL of sterile water and PB or PDB were used as quality controls. Tetracycline and/or Amphotericin B were also used as controls of growth inhibition. The microtiter plates were incubated for 18 h at 30°C; growth inhibition was determined by measuring absorbance at 595 nm.

### MICs determination

The minimal inhibitory concentration was determined using the purified peptide against the Gram-negative bacterial strains, the Gram-positive bacterial strains, the fungal strains and the yeast strains, as described above. The peptide was dissolved in sterile water and peptide concentration was measured using the method of Bradford (1976). Determination of minimal inhibitory concentrations (MICs) for Juruin was performed using a five-fold microtiter broth dilution assay of stock solution, and serial dilution in 96-well sterile plates at a final volume of 100 μL where 20 μL of stock solution was applied in to each well at serial dilution two-fold microtiter broth dilution and added to 80 μL of the bacterium/yeast dilution. Microbial growth was measured by monitoring the increase in OD at 595 nm after incubation at 30°C for 18 h (modified from Ehret-Sabatier et al., 1996). Juruin was tested in duplicate. MIC is defined as the minimal concentration of peptide that caused 100% growth inhibitions (Zhu et al., 2007). Juruin was incubated by 18 h with *C. guilliermondii* IOC 4555716, *C. krusei* IOC 4559, *C. glabrata* IOC 45658, *C. tropicalis* IOC 4560, and *Aspergillus niger* in order to identify whether is fungicidal or fungistatic by growth recovery (Baumann et al., 2010), using water, Amphotericin B and Gomesin as controls.

### Haemolytic activity

The haemolytic activity of the protein was tested using human erythrocytes. A 2.5% (v/v) suspension of washed erythrocytes in PBS was incubated with Juruin ranging from 0.125 to 10 μM in a 96-well plate for 3 h with intermittent shaking. The absorbance in the supernatant was measured at 415 nm. Haemolysis caused by PBS and 1% (v/v) Triton X-100 were used as 0% and 100% controls, respectively.

### Homology modeling

Since Juruin shows more than 60% sequence similarity to U1-theraphotoxin-Ba1a (PDB ID: 2KGH) from *Brachypelma ruhnaui*, with all of the cysteine residues conserved when aligned using Muscle (Edgar, 2004), U1-theraphotoxin-Ba1a structure, determined by NMR (Corzo et al., 2009), was used as a template for homology modeling. SWISS-MODEL (Arnold et al., 2006; Kiefer et al., 2009), an automated protein modeling server (http://www.expasy.ch/swissmod/SWISS-MODEL.html), was used to obtain a preliminary three-dimensional structural model of Juruin. However, three disulfide bonds were missing in the model. Disulfide bonds were incorporated using the Biopolymer module from Insight II software (Accelrys). The model was subjected to energy minimization using the steepest descent method (100 steps) followed by the conjugate gradient method until the RMSD (root mean square deviation) was 0.5 kcal/mol Å (1 kcal ≈4.184 kJ). The resulting structure was checked for bond length and bond angle consistency as well as peptide bond conformation. The quality of the final structure was evaluated using a Ramachandran plot.

## Results

### Purification and primary structure determination of juruin

**Figure 2:**
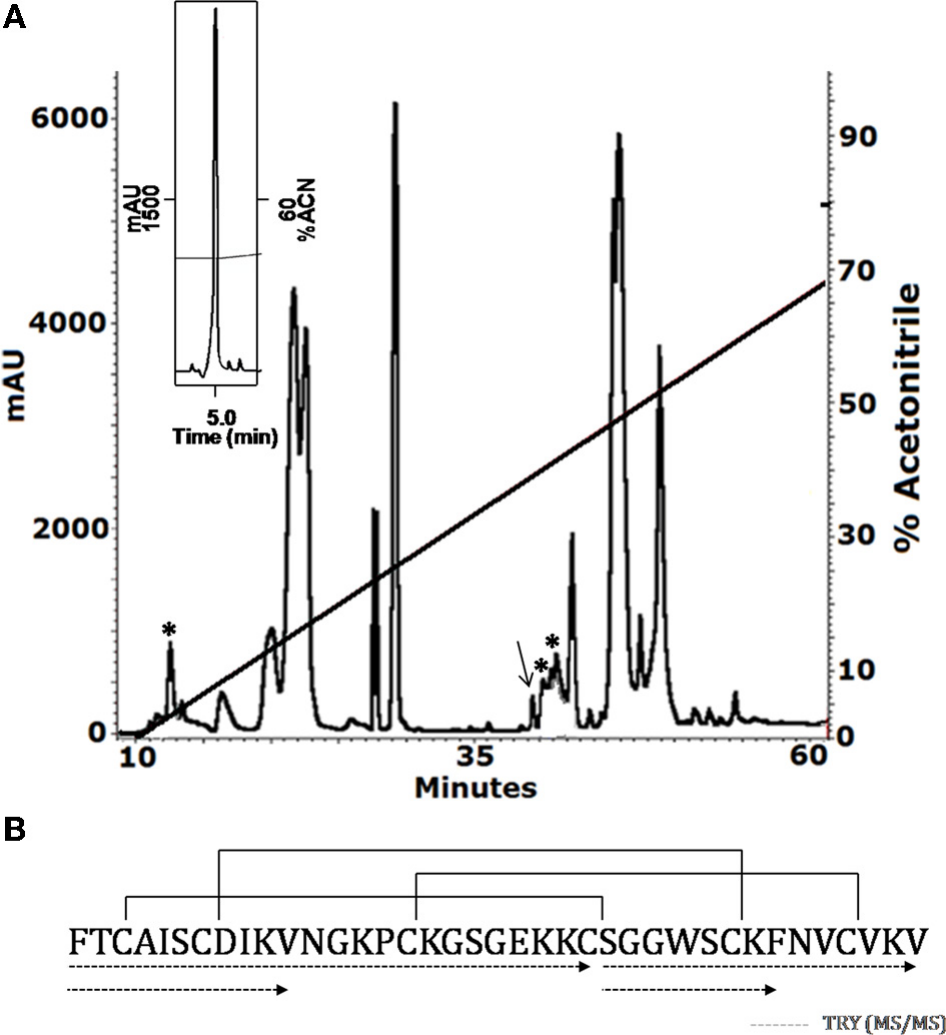
**Purification and covalent structure of Juruin. (A)** 2.5 mg of soluble venom from *A. juruensis* was separated by HPLC using a C18 reverse-phase column, eluted with a linear gradient from solution A to 80% solution B run for 50 min. The fractions labeled with the asterisk exhibited antimicrobial activity and were eluted at 11.2, 40.6, and 42.0 min, respectively. The fraction labeled with an arrow was eluted at 40.0 min, and was rechromatographed in the same system and run from 30% to 40% solution B in 60 min (inset). The major component is pure Juruin. **(B)** Complete amino acid sequence of Juruin was obtained by mass spectrometry fragmentation of several peptides obtained by enzymatic hydrolysis of Juruin, as indicated by the segments underlined by dotted lines. Solid lines linking the cysteine residues indicate the disulfide bridges in positions Cys^3^ to Cys^24^, Cys^7^ to Cys^30^, and Cys^15^ to Cys^35^. The N-terminal phenylalanine and the C-terminal amidated valine were determined by mass spectrometry (MS/MS) fragmentation.

**Figure 3:**
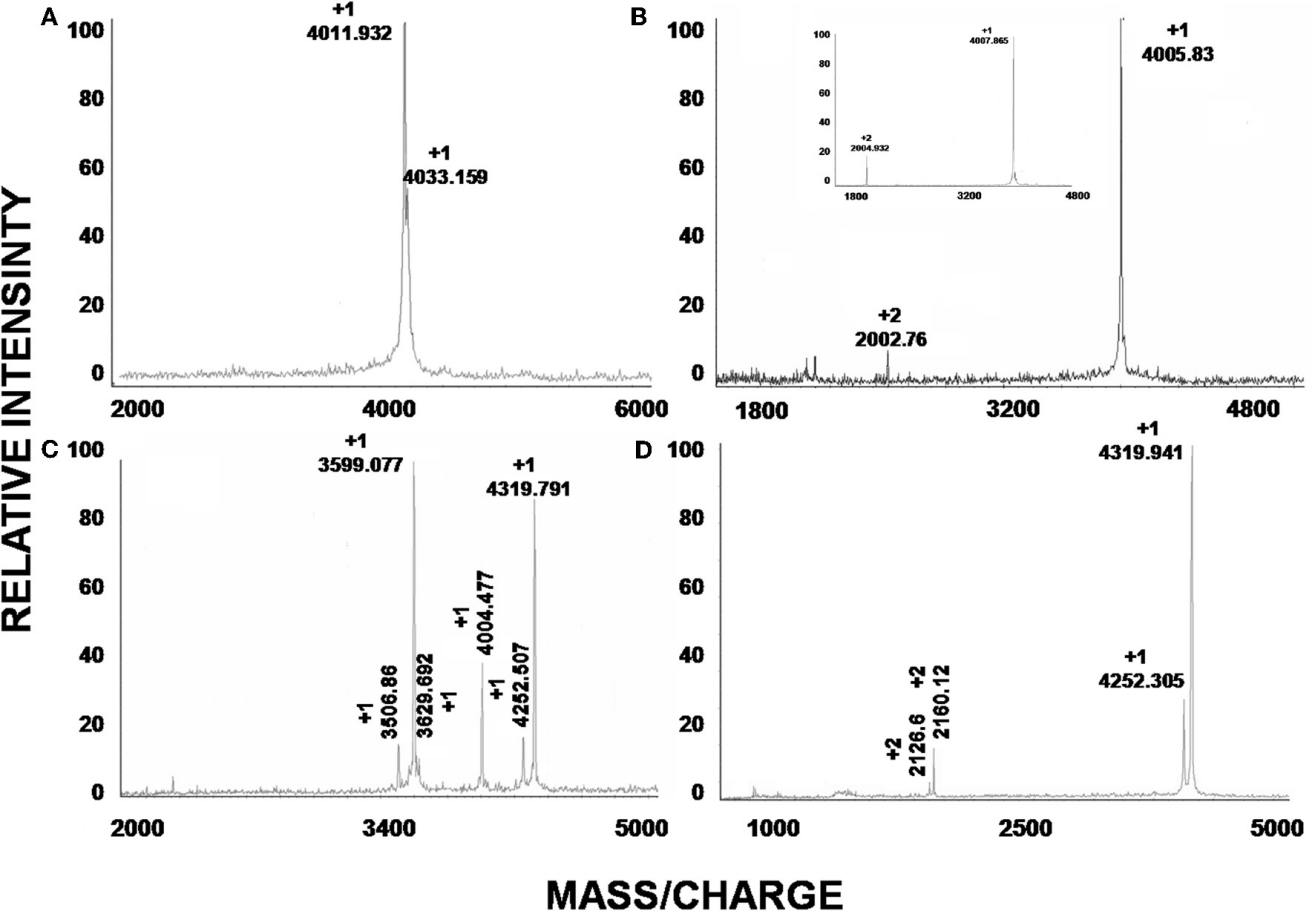
**Mass spectroscopic analysis of peptides. (A–D)** are the mass spectra of the peaks obtained by HPLC with retention times 11.2, 40.0, 40.6, and 42.0 min, respectively. **(B)** Correspond to the observed mass of native Juruin (4005.83) and reduced Juruin (inset).

The soluble venom of *A. juruensis* was separated in at least 35 different components by HPLC (Figure 2). The fraction eluted at 40.0 min, named Juruin, showed antimicrobial activity against *Candida albicans* MDM8 and was further purified until homogeneity as shown in the inset of Figure 2A. The inset graphic shows the elution of a major component (the peptide under study) plus some minor contaminants that were discarded. We have idenfied other three antimicrobial fractions, which eluted at 11.2, 40.6, and 42.0 min (Figure 2), respectively. All antimicrobial fractions were analyzed by mass spectrometry (Figure 3). Fraction eluting at 11.2 min showed two components with molecular weight of 4011.93 and 4033.16. Fraction eluted at 40.6 min showed the presence of six masses: 3506.86, 3599.07, 3629.692, 4004.47, 4252.50, 4319.79. Fraction eluted at 42.0 min showed the presence of more two components: 4252.30, 4319.94. Finally, analysis of Juruin showed the presence of a single component with a molecular weight of 4005.83 (Figure 3B). Comparision of molecular masses to previously identified spider toxins suggest these peptides contain an ICK motif (Escoubas and Rash, 2004). Therefore, we proposed the name juruentoxins for the peptides belonging to these group of toxins from *Avicularia juruensis* venom. The componentes with molecular masses: 4005.83, 4011.93, 4033.16, 3506.86, 3599.07, 3629.692, 4004.47, 4252.50, 4319.79, 4252.30, 4319.94 have been named U-theraphotoxin-Aju1a, U-theraphotoxin-Aju2a, U-theraphotoxin-Aju3a, U-theraphotoxin-AjuT4a, U-theraphotoxin-AjuT5a, U-theraphotoxin-AjuT6a, U-theraphotoxin-AjuT7a, U-theraphotoxin-Aju8a, U-theraphotoxin-Aju9a, U-theraphotoxin-Aju10a, and U-theraphotoxin-Aju11a, respectively, accordingly to a previous proposed nomenclature (King et al., 2008). Aju1a will be referred with the name Juruin, in reference to the spider *A. juruensis*, as it is the first characterized peptide from this species.

**Figure 4:**
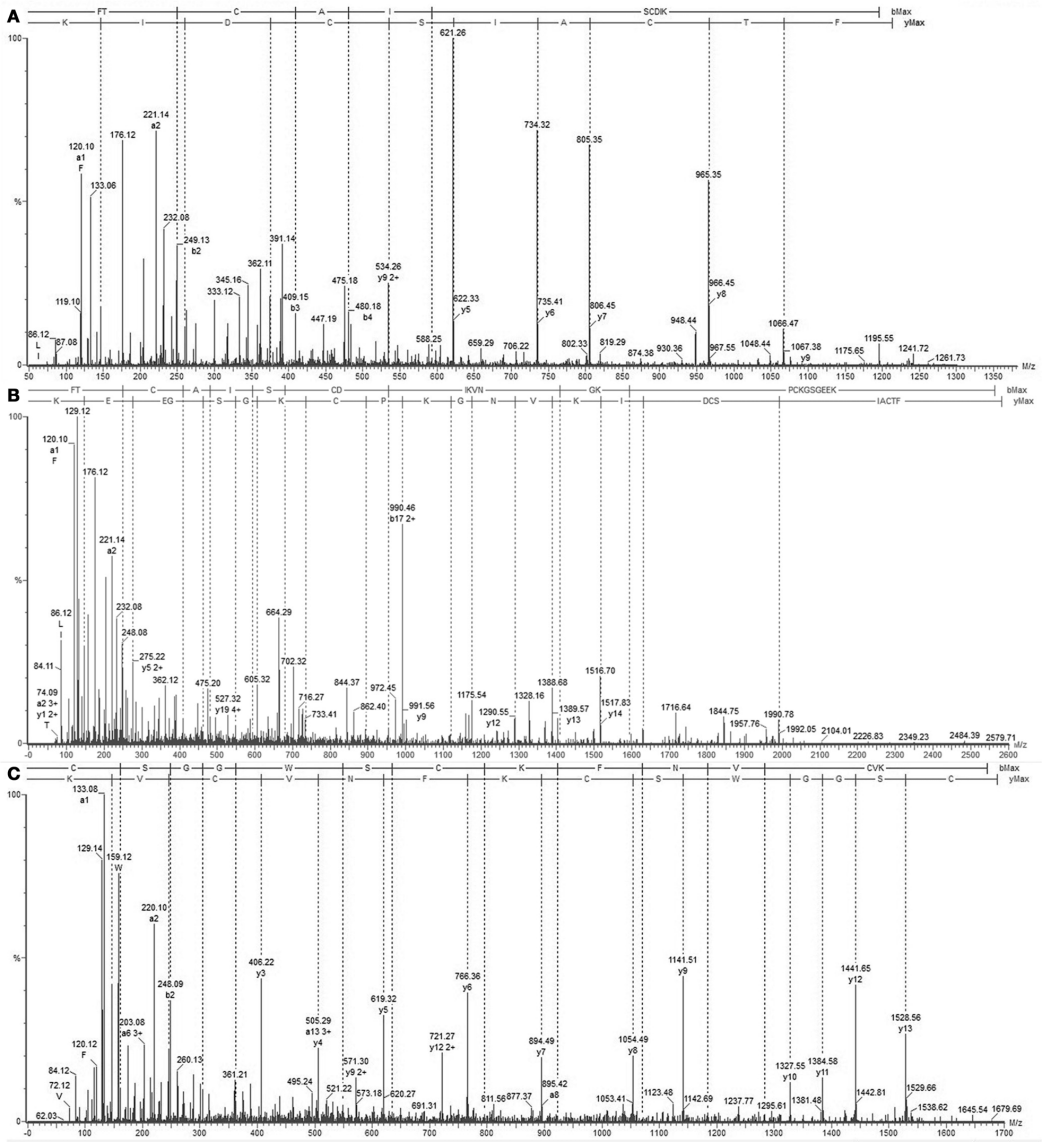
**Mass spectrometry analysis of Juruin peptides. (A)** Collision-induced dissociation spectrum from mass/charge (*m*/*z*) 1211.3 generated by trypsin digestion after analysis by LC/MS, showing the dominant fragment KIDCS with a *m/z* of 621.26, which corresponds to an N-terminal segment. **(B)** Collision-induced dissociation spectrum from *m*/*z* 2579.8, showing the b and y ion series that corresponds to the partial sequencing of the tryptic peptide between residues Phe^1^ to Lys^30^, which allowed the assignment of four cysteines, Cys^3^, Cys^7^, Cys^24^, and Cys^30^, as well as the lysine rich region Lys^22^-Lys^23^. **(C)** MS/MS spectrum from the precursor ion at *m*/*z* 1679.69 which corresponds to the C-terminus of Juruin, lacking the amidated valine at the end.

**Figure 5:**
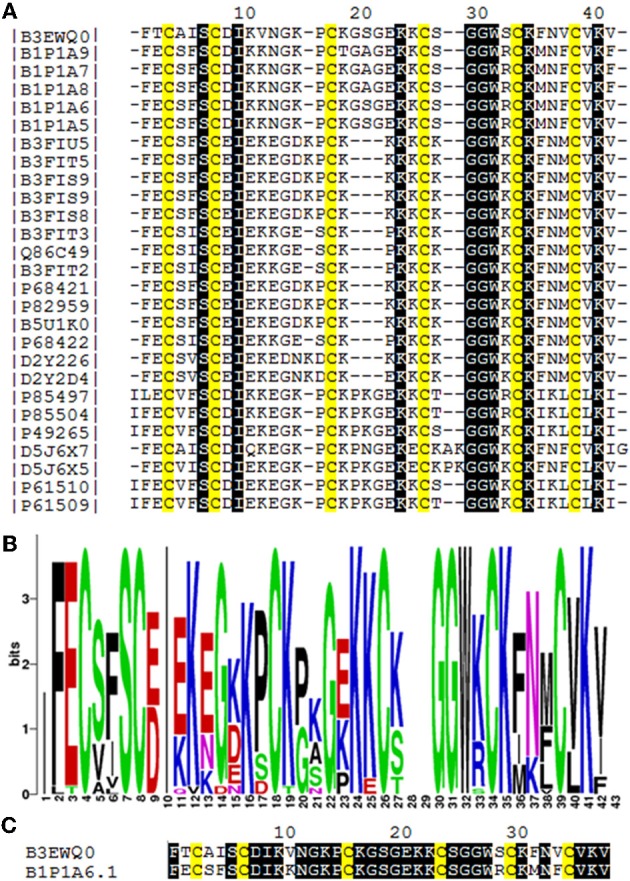
**Amino acid sequence comparision of Juruin (Aju1a) to other ICK-containing toxins. (A)** Multiple sequence alignement of Juruin to selected toxins. Amino acid sequences of toxins were retrieved from public databases and aligned with Muscle (Egdar, 2004). Cysteines are in yellow. To minimize confusion, all sequences are referred to by their UniProt accession numbers (http://www.uniprot.org/). Juruin (B3EWQ0), U3-theraphotoxin-Cj1a (B1P1A5), U3-theraphotoxin-Cj1a (B1P1A6), U3-theraphotoxin-Cj1b (B1P1A8), U3-theraphotoxin-Cj1b (B1P1A7), U3-theraphotoxin-Cj1c (B1P1A9) from *Chilobrachys jingzhao*. U1-theraphotoxin-Ba1b (P85504), U1-theraphotoxin-Ba1a (P85497) from *Brachypelma ruhnaui*. U1-theraphotoxin-Bs1a (P49265) from *Brachypelma smithi*. U1-theraphotoxin-Asp1b (P61510), U1-theraphotoxin-Asp1a (P61509) from *Aphonopelma californicum*. Putative mature sequence toxin-like RFEC (D5J6X7), putative mature peptide toxin-like LFEC (D5J6X5) from *Pelinobius muticus*. U1-theraphotoxin-Lp1b (P61506) from *Lasiodora parahybana*. Hainantoxin-II-17 (D2Y2D4), Hainantoxin-II-15 (D2Y226) from *Haplopelma hainanum*. Huwentoxin-7 (P68421), U1-theraphotoxin-Hh1a (P82959), HWTX-VIII (B5U1K0), HWTX-II (B3FIU5), HWTX-VIIIa (B3FIT5), HWTX-IIb (B3FIS9), HWTX-IIa (B3FIS8), U1-theraphotoxin-Hh1f (P68422), Huwentoxin-2a (Q86C49), HWTX-VIIb (B3FIT3), HWTX-VIIa (B3FIT2) from *Haplopelma schimidti*. **(B)** Amino acid occurrence within selected toxins. **(C)** Structural similarity of Juruin to that of U3-theraphotoxin-Cj1a, from *Chilobrachys jingzhao*, to which Juruin shares 80% sequence similiarity. Conserved residues are shown in black boxes.

We determined the amino acid sequence of Juruin by MS/MS fragmentation (Figure 4). Three main fragments were obtained after enzymatic hydrolysis with trypsin, as indicated by the dotted line under the sequence shown in Figure 2. The fragment corresponding to the positions Phe^1^ to Lys^10^ was sequenced (Figure 4A) and was further aligned with several peptides obtained by enzymatic hydrolysis, after their mass fragmentation (MS/MS) as indicated under the sequence of Figure 2. The second fragment, corresponding to the positions between Phe^1^ to Lys^23^ was sequenced (Figure 4B), positioned and correctly aligned with the previous subpeptide. The last segment, a subpeptide corresponding to positions Cys^24^ to Lys^37^, was sequenced (Figure 4C) and positioned correctly into the sequence as derived from the results of overlapping sequences obtained by mass fragmentation, as indicated. This sequence was also confirmed through the examination of another peptide, consisting of residues between Cys^24^ and Lys^31^ (data not shown). The fact that the peptide from position Phe^1^ to Lys^37^ presented a calculated molecular weight of 3907.59 Da indicates there is a missing residue at C-terminus. Considering a mass difference of 98.2 Da between the calculated mass to the mass observed by MALDI-TOF/MS suggest a valine C-amidated. Confirmation of the sequence came from the results of amino acid sequence comparison of Juruin against 91 known ICK containing peptides deposited at ArachnoServer 2.0 (Herzig et al., 2011). Whitin the Toxin-20 Family (Pfam ID: PF08089), the identities fall higher than 70% against any of the toxins considered. This high sequence similarity suggested that Juruin contains a highly conserved scaffold whitin spider toxins. In several toxins the scaffold compromises a C-terminal amidated valine (Liang, 2004). Therefore, these results suggest that Juruin has a amidated valine at the C-terminus. Additionally, sequence allignement with 26 toxins demonstrates equivalent folding of the disulfide bridges for Juruin, with disulfide pairing made between cysteines that occupy the same relative position (Figure 5). The identification of disulfide bridges are as follows: one between Cys^3^ and Cys^24^, the other between Cys^7^ and Cys^30^ and the third disulfide pairing formed by Cys^16^ to Cys^35^. The theoretically expected and the experimentally found molecular weights are similar (MW calculated: 4005.74, MW observed: 4005.83; for reduced Juruin, MW calculated: 4008.2, MW observed: 4007.8).

### Antimicrobial activity

Since many ICK toxins are reported to have antimicrobial activity, we tested native Juruin for antimicrobial activity by liquid growth inhibition assays for target pathogens, and compared with Amphotericin B and Gomesin (Silva jr. et al., 2000). Juruin showed high antimicrobial activity against all yeast and filamentous fungi tested, except for *Beauveria bassiana*. The MIC of Juruin against *C. albicans* was 2.5–5 μM. The most sensitive strains were *Candida* spp. Additionally, the filamentous fungi tested *Aspergilus niger* was highly sensitive to Juruin (MIC: 10 μM). However, Juruin did not show any antibacterial effect on the three Gram-positive strains tested, *M. luteus, S. epidermidis*, and *S. aureus*, or on the Gram-negative strains *E. coli* and *P. aeruginosa*, even at a concentration as high as 100 μM. Juruin showed similar antifungal activity to other host defense cysteine-rich peptide, Gomesin. However, when compared to Amphotericin B, the MICs are usually six fold lower than Juruin (Table 1). When *C. albicans* and *C. tropicalis* were incubated in the presence of Juruin at 20 μM for 16 h, a full growth recovery was not observed, hinting at fungicidal rather than fungistatic activity.

**Table 1:** **Antimicrobial activities of cysteine-rich antimicrobial peptides against selected microorganisms**.

**MIC [μM (μg/mL)]**	**Juruin**	**Gomesin**	**Amphotericin B**
**GRAM-POSITIVE BACTERIA**			
*Micrococcus luteus* A270	ND	0.4 (0.88)	ND
*Staphylococcus aureus* ATCC 29213	ND	1.2 (2.6)	ND
*Staphylococcus epidermidis* ATCC12228	ND	NT	ND
**GRAM-NEGATIVE BACTERIA**			
*Escherichia coli* SBS363	ND	0.4 (0.88)	ND
*Escherichia coli* ATCC 25922	ND	0.9 (1.9)	ND
*Pseudomonas aeruginosa* ATCC 27853	ND	5 (11)	ND
**FILAMENTOUS FUNGI**			
*Beauveria bassiana*	ND	NT	0.07-0.15 (0.06-0.13)
*Aspergilus niger*	5–10 (20–40)	1.2 (2.6)	0.01–0.03 (0.01–0.03)
**YEAST**			
*Candida albicans* MDM8	2.5–5 (10–20)	1.25–2.5 (2.7–5.5)	0.01–0.03 (0.01–0.03)
*Candida krusei* IOC 4559	2.5–5 (10–20)	2.5–5 (5.5–11)	0.07–0.15 (0.06–0.13)
*Candida glabrata* IOC 45658	2.5–5 (10–20)	>10 (22)	ND
*Candida albicans* IOC 45588	2.5–5 (10–20)	5–10 (11–22)	ND
*Candida parapsilosis* IOC 456416	2.5–5 (10–20)	2.5–5 (5.5–11)	0.07–0.15 (0.06–0.13)
*Candida tropicalis* IOC 45608	2.5–5 (10–20)	0.3–0.6 (0.6–1.2)	0.07–0.15 (0.06–0.13)
*Candida guilliermondii* IOC 455716	2.5–5 (10–20)	2.5–5 (5.5–11)	0.07–0.15 (0.06–0.13)

The activity of Juruin was compared to Gomesin (Silva jr. et al., 2000) and Amphotericin B. MIC values (μM) refer to the minimal inhibitory concentration required toachieve 100% growth inhibition. ND, not detected in the ranges assayed; NT, not tested.

### Haemolytic activity

To investigate whether Juruin has any effect on mammalian membranes at the antimicrobial concentration range, its haemolytic effect was tested. After incubating human erythrocytes with the protein up to 10 μM concentrations, no haemoglobin release was observed, indicating that Juruin does not cause lysis of erythrocyte membrane within these concentrations (results not shown).

### Structure-function relationship studies

**Figure 6:**
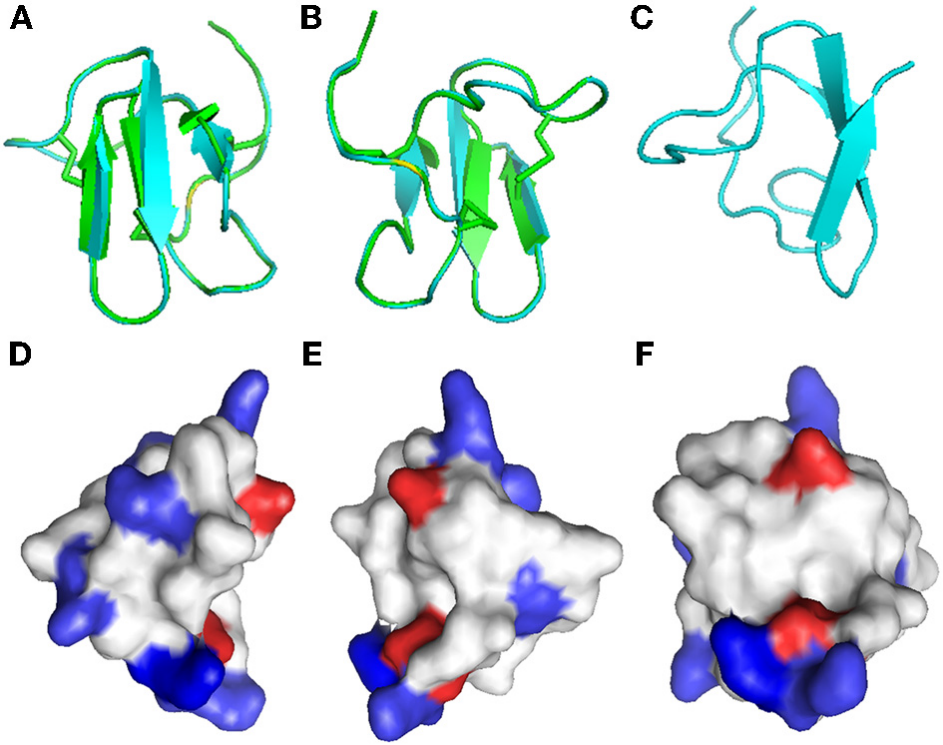
**Homology modeling of Juruin.** Comparison of Juruin and U1-theraphotoxin-Ba1a (PDB ID: 2KGH), from *Brachypelma ruhnaui*, structures. **(A,B)** Juruin (blue) and U1-theraphotoxin-Ba1a (green) ribbon structures were superimposed over the backbone atoms. **(C)** Ribbon structure of Juruin in a different view related by ~90° rotation. **(D,E)** Molecular surface of Juruin highlighted to show electrostatic potential, surfaces with positive, negative, and neutral electrostatic potentials are drawn in blue, red, and white, respectively. **(F)** The Lys^22^-Lys^23^ segment shows maximum positive charge localization represented by the intense blue color. Model pairs show the sides of the protein rotated by ~180°.

Disulfide bridges are required for the highly compact, stabilized folding of many cysteine-rich proteins and their biological function, such as the antimicrobial properties of β-defensins (Yenugu et al., 2003). Due to the lack of material, we could not determine the importance of disulfide bonds and folding for the antifungal properties of Juruin. Instead, a three-dimensional model of Juruin was built using the known structure of U1-theraphotoxin-Ba1a (PDB ID: 2KGH), from *Brachypelma ruhnaui* (Corzo et al., 2009), since it shows 60% identity, with all of the cysteine residues conserved. The homology model of Juruin is shown in Figure 6. The final structure had φ and ψ angles within the allowed region of the Ramachandran map and all the peptide bonds were *trans*. A total of 15 residues (39.47%) were in the fully allowed region, 11 residues (28.95%) were in the additionally allowed region, nine residues (23.7%) were in the generously allowed region and only three residues (7.9%) were in the outside region. Juruin model shows similarity to the structure of U1-theraphotoxin-Ba1a, consisting of an ICK motif with three cross-linked disulfide bonds. The structure consists of three antiparallel β-sheet at residues 14–16, 27–31, and 34–37. The remaining part of the molecule is loop-structured (Figures 6A–C). The analysis of the electrostatic potential of the molecule reveals that the charge distribution is distinct, in which the middle segment of the molecule harbors maximum surface positive charge (Figure 6F), which may be essential for antimicrobial activity (Yin et al., 2012). This region, between Cys^16^ to Cys^24^ of Juruin, comprises three positively charged residues out of nine residues (CKGSGEKKC).

## Discussions

The findings described in this study provide novel information for the development of antimicrobial drugs. We have described the isolation and the complete covalent structure determination of Juruin. To our knowledge, this is the first peptide isolated from *A. juruensis*, a mygalomorph spider which belongs to the Theraphosidae family (Figure 1). When we analyzed the structure of Juruin in the context of what is known for the other spider toxins (Escoubas and Rash, 2004; Kuhn-Nentwig et al., 2004; Liang, 2004), it is clear that its structure is highly conserved. However, we could identify six novel residues within a highly conserved scaffold from an arboreal spider toxin (Figure 5C). We suggest that these residues might have been positively selected during evolution, which can cause an increase in the binding affinity to its target receptor.

Firstly, we have screened the venom of *A. juruensis*, monitoring the antimicrobial activity using liquid growth inhibition assays. Out of more than 40 fractions, we have identified only four fractions which exhibited toxicity to tested microorganisms (Figure 2). Mass spectrometric analysis revealed the masses of 11 compounds: 4005.83, 4011.93, 4033.16, 3506.86, 3599.07, 3629.692, 4004.47, 4252.50, 4319.79, 4252.30, 4319.94 (Figure 3), which have been named U-theraphotoxin-Aju1a, U-theraphotoxin-Aju2a, U-theraphotoxin-Aju3a, U-theraphotoxin-AjuT4a, U-theraphotoxin-AjuT5a, U-theraphotoxin-AjuT6a, U-theraphotoxin-AjuT7a, U-theraphotoxin-Aju8a, U-theraphotoxin-Aju9a, U-theraphotoxin-Aju10a, and U-theraphotoxin-Aju11a, respectively, accordingly to a previous proposed nomenclature (King et al., 2008). Aju1a will be referred with the name Juruin, in reference to the spider *A. juruensis*, as it is the first characterized peptide from this species.

When compared to other known toxins from spiders, these compounds shows similarity to ICK-containing peptides, with molecular masses ranging from 3.0 kDa to 7.5 kDa (Escoubas and Rash, 2004). ICK-containing peptides in spider venom seem to have evolved from β-defensins, while they have gained novel functions during evolutionary recruitment events (Fry et al., 2009). Therefore, even highly specific neurotoxins have been shown to exhibit a high antimicrobial activity (Kuhn-Nentwig, 2003), and also antimicrobial compounds at spider venoms act in synergism to neurotoxins (Kuhn-Nentwig et al., 2004). In this work, we investigated the antimicrobial properties from the compound with 4,005.83 Da, named Juruin, which has been purified to homogeneity (Figure 2). Further research will focus on those other compounds.

Juruin is a 38-residue peptide with three disulphide bridges conformed in an ICK motif, and a valine amidated at the C-terminus, similarly to previous identified huwentoxins from *Selenocosmia* (*Ornithoctonus*) *huwena* (Liang, 2004). The disulfide bridges and C-terminal amidation certainly contributes to the stability of the peptide to proteases within the venom or when it is released for defense or against a prey (Silva jr. et al., 2000). Juruin is a highly cationic AMP, with seven positively charged residues (seven Lys) with a calculated pI of 9.08. Our structural model and sequence allignment suggest that the six cysteine residues in Juruin form three disulfide bridges linking Cys^3^-Cys^24^ (CI-CIV), Cys^7^-Cys^30^ (CII-CV), and Cys^16^-Cys^35^ (CIII-CVI), such disulphide array is identical for all ICK-containing toxins from spiders (Escoubas and Rash, 2004). Interestingly, in Juruin an equal number of residues is observed between disulfide bridges as it is observed among other ICK-containing toxins (Figure 5A). Sequence comparison between Juruin to U3-theraphotoxin-Cj1a, from *Chilobrachys jingzhao*, to which Juruin shares 80% sequence similarity, reveals the difference of only six residues (Figure 5C). It has more than 70% of sequence similarity to Toxin-20 family (Pfam ID: PF08089) of peptides, from the spiders *Ornithoctonus huwena* (Liang, 2004; Yuan et al., 2007; Jiang et al., 2010), *Chilobrachys jingzhao* (Liao et al., 2007; Chen et al., 2008) and *Haplopelma hainanum* from China (Pan and Yu, 2010; Tang et al., 2010), which are neurotoxins with broad biological activities, including: voltage-gated ion channels inhibition, bioinsecticidal activity and inhibition of trypsin (Liang, 2004). While the three residues between Cys^3^-Cys^7^ (Ala^4^, Ile^5^, Ser^6^) have already been identified in the putative mature sequence toxin-like RFEC (Uniprot ID: D5J6X7) from *Pelinobius muticus*, Thr^2^, Val^11^, Ser^29^, Phe^32^, and Val^34^ are novel residues within this toxin scaffold. When compared to more than 91 toxins, none of them presents those residues at these positions. It is likely that these amino acids residues are important to bioactivity of Juruin, being positively selected during evolution, instead of having appeared from neutral mutations. Surprisingly, Juruin is the unique Toxin-20 Family belonging peptide which has a Thr^2^ at the N-terminal region. In contrast to all species producing Toxin-20 family proteins, which are burrowing tarantulas, *A. juruensis* is an arboreal spider from the Amazonian rainforest also known as bird eating tarantula, because it often prey small vertebrates such as birds, small lizards, and tree frogs. Therefore, higher toxicity against vertebrates as well as unusual activities in the venom of an arboreal species could have appeared from divergent sequence patterns and positive Darwinian selection. The Brazilian insular arboreal pitviper *Bothorps insularis* (Cogo et al., 1998) show variation on the snake venom composition as well as divergent sequence patterns which might be the result of a significant dietary habit change and positive Darwinian selection causing an increase in venom toxicity. Therefore, Juruin should be included in further phylogenetic analyses with other ICK containing toxic peptides.

Juruin is effective against the majority of the fungi and yeast strains tested, with MICs between 2.5–5 μM for all of them, except for *Aspergilus niger* which showed MIC between 5–10 μM. Although cysteine-rich AMPs play an important role on spider immune system and often show a broad spectrum of activity against pathogens (Silva jr. et al., 2000), Juruin, a highly knotted cysteine-rich AMP, didn't show antibacterial activity against Gram-positive and Gram-negative bacteria tested. Also, antifungal activity against *B. bassiana* could not be observed. Juruin has marked activity against a variety of yeast at a rather low concentration, the most resistant strain being the yeast *C. glabrata* (Table 1). Interestingly, Juruin has similar MICs to that of Gomesin, a potent host defense peptide previously identified by our group (Silva jr. et al., 2000). Amidated Gomesin has a slightly more pronounced active antimicrobial effect when compared to that of non-amidated form. The lack of disulphide pattern in Gomesin after reduction/alkylation produce a decrease in antimicrobial activity. Similarly, disulphide bridge pattern and post-translational modification might be related to antimicrobial activity and a putative neurotoxic effect of Juruin. However, Amphotericin B is effective even in six-fold lower concentrations (μM) than that of Juruin. On the other hand, Juruin is effective against Amphotericin B-resistant strains, *C. albicans* IOC 45588 and the clinically important *C. glabrata* (Krogh-Madsen et al., 2006; Khan et al., 2008).

We tested the toxicity to human erythrocytes only within the antimicrobial ranges. Juruin do not show haemolytic activity even at the higher concentration tested 10 μM. This data suggests that the mode of action of Juruin is not by disrupting cell membranes. Moreover, the presence of a large number of positively charged amino acids in host defense peptides contributes to a higher specificity of the peptide to a higher electronegative charged targets, such as prokaryotic cells (Silva jr. et al., 2000), nucleic acids or intracellular proteins (Nguyen et al., 2011). Therefore, the positively charged residues (Figure 6) might be involved in target receptor recognition and selectivity against pathogens and preys.

The specific antimicrobial activity of Juruin against fungi and yeast gives novel evidences for the origin of arachnid toxins from antifungal β-defensins (Zhu et al., 2005). After one or several recruitment events, defensins sequence duplication and wide divergence driven by positive Darwinian selection might have expanded this class of molecules into new functional groups (Fry et al., 2009), probably including ICK-containing toxins. The evidence of positive Darwinian selection in the ICK fold within spider toxins suggests that adaptive amino acid changes in a conserved scaffold are a major force driving new functional emergence. Therefore, divergent sequence patterns should be used for peptide-based drug design (Zhu et al., 2005, 2011). Hence, the identification of novel residues within a highly conserved scaffold offers a potential to investigate the divergent evolution of *A. juruensis* toxins, and Juruin is a natural template for development of novel therapeutical drugs.

In summary, we have isolated, purified and characterized a new ICK-containing AMP, named Juruin (Aju1a). The remarkable similarity to other toxins with other interesting bioactivities such as ion channels modulations (Liang, 2004) and antiparasitic activity (Pimentel et al., 2006), the highly conserved primary structure of the toxin, along with its selectivity, potent fungicidal activity, and the lack of haemolytic activity against human erythrocytes together with a putative resistance against proteases, makes Juruin pharmacologically interesting and valuable for the design of novel efficient drugs against fungal diseases. Thus, Juruin unique sequence should be investigated as a novel prototype for drug development.

### Conflict of interest statement

The authors declare that the research was conducted in the absence of any commercial or financial relationships that could be construed as a potential conflict of interest.
